# Ultrasonic-Assisted Extraction of Flavonoids from *Juglans mandshurica* Maxim.: Artificial Intelligence-Based Optimization, Kinetics Estimation, and Antioxidant Potential

**DOI:** 10.3390/molecules27154837

**Published:** 2022-07-28

**Authors:** Guodong Chu, Rui Liang, Chenmeng Wan, Jing Yang, Jing Li, Ruinan Wang, Linna Du, Ruixin Lin

**Affiliations:** 1 Ministry of Education Engineering Research Center of Bioreactor and Pharmaceutical Development, College of Life Science, Jilin Agricultural University, Changchun 130118, China; cgd0429@163.com (G.C.); wcm159357@163.com (C.W.); yangjing5122010@163.com (J.Y.); lijing5372@163.com (J.L.); wangruinan98@163.com (R.W.); 2 Engineering Research Center of Chinese Ministry of Education for Edible and Medicinal Fungi, College of Plant Protection, Jilin Agricultural University, Changchun 130118, China; l18890898222@163.com; 3 Department of Hepatobiliary and Pancreatic Surgery, The Second Hospital of Jilin University, Jilin University, Changchun 130041, China

**Keywords:** ultrasonic-assisted extraction, *Juglans mandshurica* Maxim., flavonoids, artificial neural network, antioxidant

## Abstract

Ultrasonic-assisted extraction (UAE) of flavonoids (JMBF) from *Juglans mandshurica* Maxim., an important industrial crop in China, was investigated in the present study. To improve the extraction efficiency of JMBF, suitable UAE was proposed after optimization using a hybrid response surface methodology–artificial neural network–genetic algorithm approach (RSM–ANN–GA). The maximum extraction yield (6.28 mg·g^−1^) of JMBF was achieved using the following optimum UAE conditions: ethanol concentration, 62%; solid–liquid ratio, 1:20 g·mL^−1^; ultrasonic power, 228 W; extraction temperature, 60 °C; extraction time, 40 min; total number of extractions, 1. Through the investigation of extraction kinetics, UAE offered a higher saturated concentration (*C_s_*) for JMBF in comparison to traditional solvent extraction (TSE). Scanning electron microscopy (SEM) images showed that deeper holes were generated in *J. mandshurica* powder under the action of ultrasound, indicating that ultrasound significantly changed the structure of the plant materials to facilitate the dissolution of active substances. Extracts obtained using UAE and TSE were compared by Fourier-transform infrared spectroscopy analysis, the results of which revealed that the functional group of bioactive compounds in the extract was unaffected by the ultrasonication process. Moreover, JMBF was further shown to exhibit significant antioxidant properties in vitro. This study provides a basis for the application of JMBF as a natural antioxidant.

## 1. Introduction

Natural products, including traditional medicinal plants and their extracts, have a long history in the treatment of human diseases, leading to the increasingly prominent role of plants in potential drug discovery [1,2]. According to historical records, more than 700 plant-derived products have been used for medical treatments [3]. *Juglans mandshurica* Maxim., a widely distributed walnut species, has received extensive attention due to its beneficial effects on health [4]. Modern research shows that *J. mandshurica* has various activities including antibacterial, antitumor, and anti-inflammatory properties [5] Interestingly, the medical and health protection value of each part of *J. mandshurica* has been reported, and its bark has attracted increasing attention due to its large number of active ingredients [6]. Among the various active components of this plant, flavonoids are considered as indispensable substances in the fields of nutrition and pharmacy [7,8]. Although *J. mandshurica* has been widely studied, most of the studies focused on anthraquinones, naphthoquinones, and other components, whereas little research has been carried out on its total flavonoids [9].

Flavonoids, low-molecular-weight polyphenolic compounds, are the main bioactive secondary metabolites of *J. mandshurica* [10]. Flavonoids have shown prominent effects in a variety of acute and chronic human diseases, which are closely related to their antioxidant, immunomodulatory, and other properties [11]. Meanwhile, flavonoids also demonstrate a protective effect on cancer, with potential for use as chemotherapeutic agents [12]. Moreover, various studies have shown that the number of side-effects of flavonoids obtained from plants is very low [13]. Thus, with the many medicinal benefits of flavonoids, it seems that the extraction and the utilization of total flavonoids from *J. mandshurica* are necessary. However, as reported previously, the amount of total flavonoids is closely related to its activity [14]. Therefore, the establishment of an appropriate extraction process is particularly important for the further development of flavonoids from *J. mandshurica*.

Traditionally, various extraction technologies have been proposed and used for the preparation of flavonoids from plants, including boiling, refluxing, and soaking. However, some shortcomings have limited the application of these techniques, such as the large amount of high-purity organic solvents required, lower extraction efficiency, and longer extraction time [15,16]. In recent years, the green extraction technology has attracted more and more attention due to its characteristics, including high extraction efficiency, low energy consumption, and lower amounts of organic solvent required [17,18]. Ultrasound-assisted extraction (UAE) has been considered one of the most popular green extraction technologies, due to its acoustic cavitation, thermal effects, and mechanical effects [19,20]. Hence, to promote the utilization of *J. mandshurica*, UAE was employed for the extraction of total flavonoids in the present study.

The extraction conditions are an important part of maximum efficacy. Many reports have shown that multiple factors influence the extraction efficiency of UAE; thus, it is necessary to obtain the best extraction conditions of total flavonoids from *J. mandshurica* in a short time [21]. Over the past several years, artificial intelligence-based optimization methods, including artificial neural network (ANN) and genetic algorithm (GA), have been considered faster and more cost-effective approaches to solve optimization problems [22]. The introduction of artificial intelligence-based tools has greatly reduced the number of required experiments and has led to a reduction in reagent consumption and laboratory work [23]. Moreover, artificial intelligence-based tools also play an important role in the statistical significance analysis of the effect of selected factors on response values, as well as the interaction effects between independent variables [24]. Thus, artificial intelligence-based tools have been frequently used to help optimize systems in many fields such as food, environment, and pharmacy [25].

Various artificial intelligence-based approaches were employed to optimize the suitable UAE conditions for the extraction of total flavonoids from *J. mandshurica* (JMBF) in the present study. Subsequently, a variety of comparative analyses on UAE and traditional solvent extraction (TSE) for JMBF were carried out. Finally, the antioxidant potential of JMBF obtained by UAE was studied further.

## 2. Results

### 2.1. Selection of Optimal Ultrasonic Extraction Parameters of JMBF by Single-Factor Test

**Figure 1:**
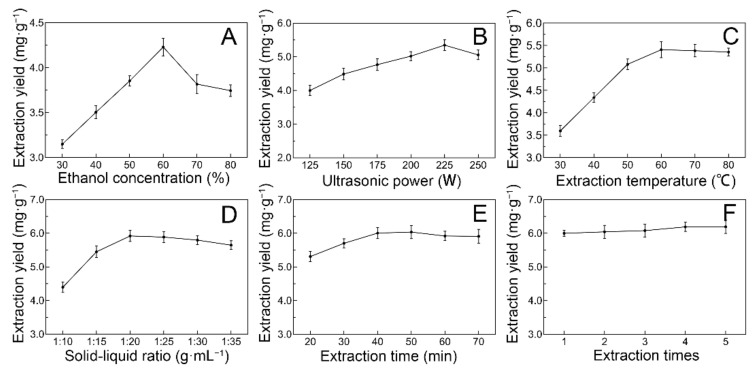
The effects of different ethanol concentration (**A**), ultrasonic power (**B**), extraction temperature (**C**), solid–liquid ratio (**D**), extraction time (**E**), extraction times (**F**) on the extraction yield of flavonoids.

The effects of different ultrasonic extraction parameters on the extraction yield of total flavonoids from *J. mandshurica* (JMBF) were assessed using a single-factor test (Figure 1). As illustrated in Figure 1A, the extraction yield of total flavonoids was directly proportional to the ethanol concentration in the range of 30% to 60%. The highest extraction efficiency of JMBF was achieved when the ethanol concentration was 60%. Subsequently, the extraction yield of flavonoids decreased gradually with the continuous increase in the percentage of ethanol in the extraction solvent. It was speculated that ethanol with a concentration higher than 60% would accelerate the dissolution of some other alcohol-soluble and lipophilic impurities, thus reducing the solubility of the flavonoids in the solvent. Therefore, 60% ethanol was used as the extract solvent in the subsequent experiments. Similarly, the optimal ultrasonic power, extraction temperature, solid–liquid ratio, and extraction time were 225 W, 60 °C, 1:20 g·mL^−1^, and 40 min, respectively (Figure 1B–E). Furthermore, the effect of extraction times on the response value was also considered (Figure 1F). The results implied that multiple extractions did not markedly improve the response value. Thus, considering the cost, the best number of extraction times for JMBF was one.

### 2.2. Contribution of Different Parameters to the Extraction Yield of JMBF

The Plackett–Burman design (PBD) test was employed to identify the factors that significantly influence the extraction yield of JMBF. Ethanol concentration (%, X_1_), extraction temperature (°C, X_2_), extraction time (min, X_3_), ultrasonic power (W, X_4_), and solid–liquid ratio (g·mL^−1^, X_5_) were included in the scope of investigation, while other variables were virtual items. In the 12 groups of experiments, the variation range of response values was large, and it was speculated that the conditions of ultrasound-assisted extraction (UAE) have a strong influence on the yield of flavonoids (Table 1). The data were analyzed using SAS V8 software, and the following multivariate first-order equation with a determination coefficient of 0.97 was obtained:

Y = 5.0680 + 0.3070X_1_ + 0.6033X_2_ − 0.0260X_3_ − 0.2699X_4_ + 0.1494X_5_ − 0.1059X_6_ + 0.0497X_7_ − 0.0668X_8_ (1)

**Figure 2:**
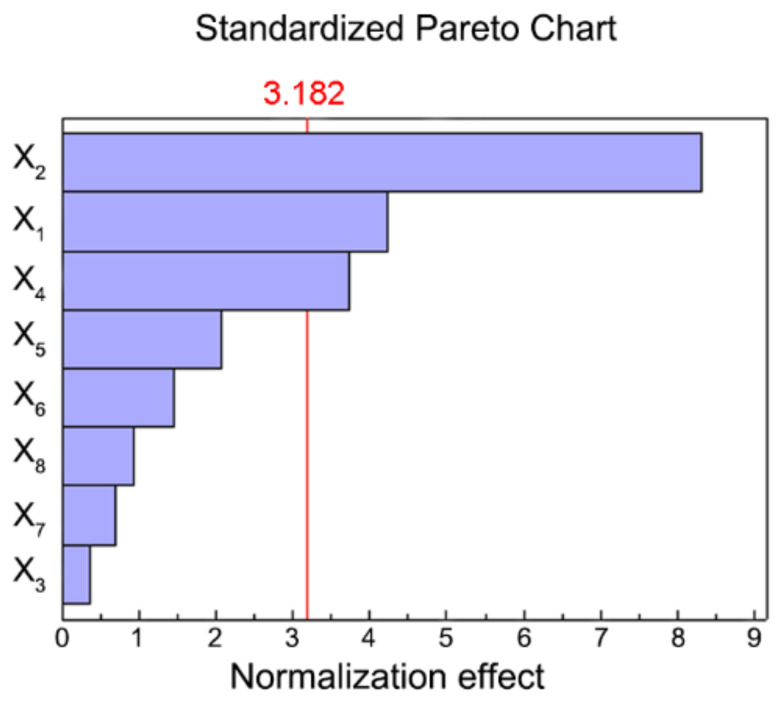
Pareto plot of standardized effects of tested variables on the extraction yield of flavonoids. The red line represents a 95% confidence interval, and exceeding this reference line indicates that the factor has a significant effect on the response value.

F-test analysis was introduced, proving that the above model was significant (*p* < 0.05). By regression analysis, the importance of each investigation factor in the process of UAE was found to be X_2_ > X_1_ > X_4_ > X_5_ > X_6_ > X_8_ > X_7_ > X_3_, in which X_2_, X_1_, and X_4_ were significant items (*p* < 0.05) (Table A1). A Pareto plot also reflected that the variables X_2_, X_1_, and X_4_ were the three factors most affecting the extraction yield of JMBF (Figure 2). In summary, extraction temperature (X_2_), ethanol concentration (X_1_), and ultrasonic power (X_4_) were selected for further optimization.

### 2.3. Optimization of the Significant Parameters Using Response Surface Methodology and Artificial Neural Network–Genetic Algorithm

The Box–Behnken design (BBD) test was used to optimize the values of key factors, the results of which are displayed in Table 2, and a multivariate quadratic regression (MQR) was used to establish a fit, as follows:

Y = 6.1889 + 0.0699X_1_ + 0.2457X_2_ + 0.0097X_3_ − 0.2391X_1_^2^ + 0.0598X_1_X_2_ + 0.0501X_1_X_3_ − 0.3280X_2_^2^ + 0.0104X_2_X_3_ − 0.6114X_3_^2^ (2)

**Figure 3:**
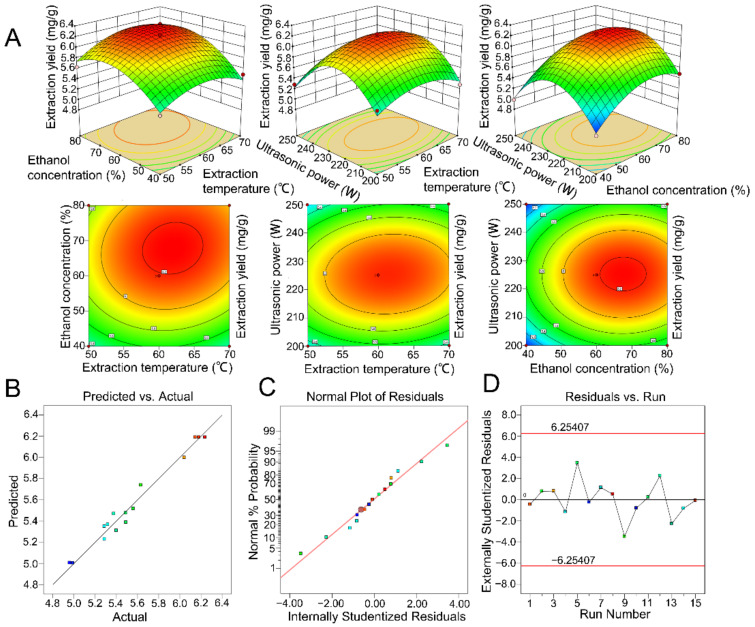
Results of the response surface analysis. (**A**) Response surface plots and contour plots illustrating the interactive effects of different variables on the yield of flavonoids. The different colors in the figure represent the response values predicted by the model with different independent variables. When the predicted response value increases from small to large, the color of the response surface gradually changes from blue to red. (**B**) Plot of predicted vs. observed values. (**C**) Plot of normal probability vs. internally studentized residuals. (**D**) Plot of studentized residual vs. run number.

The fitting degree of this model was good, and there was a high correlation between the predicted value and the experimental value (determination coefficient *R*^2^ = 0.9751). According to the *p*-value of the model, the MQR model was reliable (*p* < 0.05). Through the regression analysis of the results, it was found that there was no simple linear relationship between three variables and the response variable, in which the *p*-values of the linear term (X_2_) and quadratic term (X_1_^2^, X_2_^2^, X_3_^2^) were less than 0.05 (Table 3). These results were also consistent with the results of the response surface and contour plots (Figure 3A). Figure 3B shows the positive correlation between the observed and predicted values. The proximity of values on both sides of the line indicates a strong correlation between the model data and the actual data.

The variation of the normal percentage probability to the residual is illustrated in Figure 3C. All the residuals were linear relative to the normal percentage probability, indicating the rationality of the model prediction and confirming the assumption that the error term was a normal distribution. Additionally, the studentized residual versus the run number was plotted. As portrayed in Figure 3D, no point was outside the red boundary, indicating that there was no potential outlier, and the errors of all points were within the allowable range.

**Figure 4:**
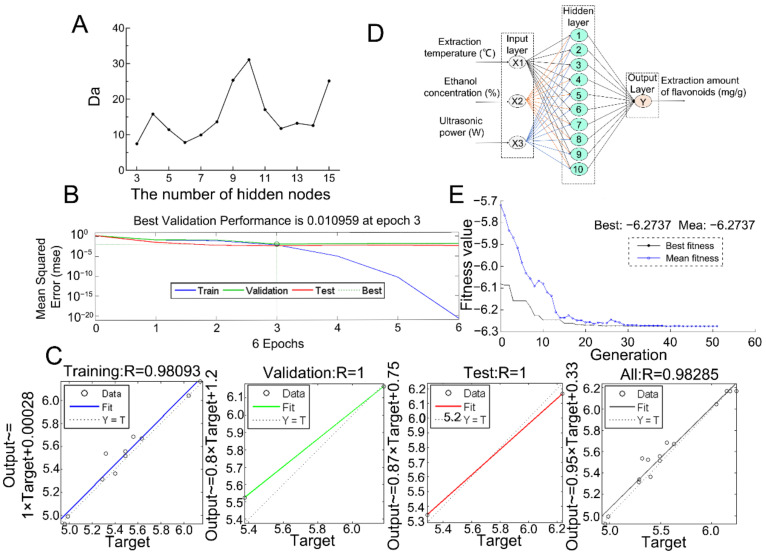
ANN modeling and training. (**A**) The effect of the number of neurons in the hidden layer on the degree of approximation (*Da*). (**B**) Training performance of ANN. (**C**) Regression analysis diagram of actual data and predicted data by ANN (3–10–1) model. (**D**) ANN model architecture topology for JMBF extraction (3–10–1). (**E**) Evolution of the best and average fitness in the genetic algorithm.

The data obtained from the BBD experiments were randomly selected as a test set (70%), prediction set (15%), or calibration test (15%), and a three-layer artificial neural network (ANN) model was constructed. The proper number of hidden nodes was selected by degree of approximation (*Da*) and set to 10 (Figure 4A). The performance of the validation error (MSE) during training, validation, and testing with respect to the increase in the epoch is shown in Figure 4B. It was found that the best validation performance was obtained at three epochs with an MSE value of 0.0110. Figure 4C presents the regression analysis between ANN predicting data and the experimental values. The correlation coefficient (*R*) of the overall ANN model for all datasets was 0.98, which indicated good agreement between ANN data and the actual data. These results justified the reliability of the developed ANN model for the prediction of the UAE process. Hence, the final optimum ANN architecture shown in Figure 4D was chosen as the input to generate the best fitness function for optimizing the extraction conditions using a genetic algorithm (GA).

The best fitness plot for the GA is shown in Figure 4E. With the increase in generations, the fitness value decreased gradually, and the GA stopped selecting when the generation was 50. The software analysis showed that the best coded values for the three factors were X_1_ = 0.027, X_2_ = 0.119, and X_3_ = 0.143, respectively, and the corresponding actual values were x_1_= 60 °C, x_2_= 62%, and x_3_ = 228 W, respectively. It was predicted that the maximum extraction yield of JMBF was 6.2738 mg·g^−1^. Five validation experiments were carried out to evaluate the reliability of the model prediction results. The average extraction yield of flavonoids was 6.28 mg·g^−1^, and there was a 0.099% relative error between it and the predictive values. These results suggested that artificial intelligence-based approaches were feasible and stable for the optimization of the UAE process for the flavonoids.

### 2.4. Kinetic Study of Bath Ultrasonic-Assisted Extraction and Traditional Solvent Extraction Procedure

Batch UAE and traditional solvent extraction (TSE) for JMBF were implemented under the optimal conditions, and the extraction yield of the flavonoids in the extracts at different extraction times were detected. Graph Pad Prism 8 software was introduced to fit the obtained data from various batch experiments, and the values of *C_s_* and *k* were calculated using the formula below, which was obtained by integrating Equation (5).

(3) $$\frac{t}{C_{t}}=\frac{1}{KC_{s}^{2}}+\frac{t}{C_{s}}$$

where *t* represents the extraction time (min). *C_t_* and *C_s_* denote the concentration of flavonoids at time *t* and the saturated concentration of flavonoids, respectively. The rate constant for extraction is *K*.

**Figure 5:**
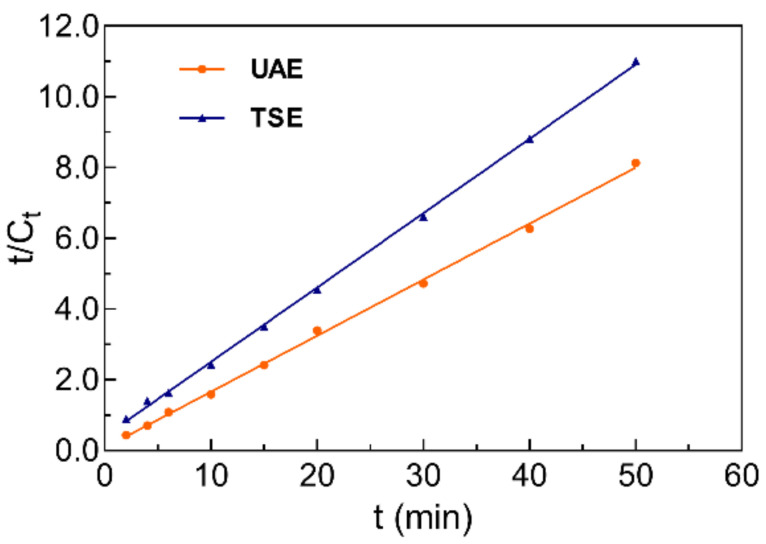
The relationship of *t*/*C_t_* vs. *t* from batch experimental extraction data of UAE and TSE.

The results showed that the second-order model with high determination coefficients was suitable for explaining the extraction process of flavonoids by UAE (0.9985) and TSE (0.9994) (Figure 5). A linear relationship between t and *t*/*C_t_* was found, and the reciprocal of the linear slope was the saturated concentration (*C_s_*). The *C_s_* value for JMBF obtained by UAE was calculated to be 6.31 mg·g^−1^, and the relative error was 0.57% from the previous predicted value. However, the *C_s_* value of JMBF measured by TSE was only 4.76 mg·g^−1^, a 24.7% reduction in *C_s_* value compared with UAE. The second-order rate constant (*k*) was calculated by the intercept of the straight line, and the *k*-values of UAE and TSE were calculated to be 0.33 and 0.11, respectively. These results showed that UAE has a higher rate constant than TSE.

### 2.5. Comparison of the Optimal UAE with TSE Procedure

Several comparative analyses of the proposed UAE and TSE were performed to further evaluate the extraction capacity of UAE for natural products, including scanning electron microscopy (SEM) observation and Fourier-transform infrared spectroscopy (FTIR) analysis.

#### 2.5.1. Microscopic Structures of *J. mandshurica* Treated with Different Extraction Techniques

**Figure 6:**
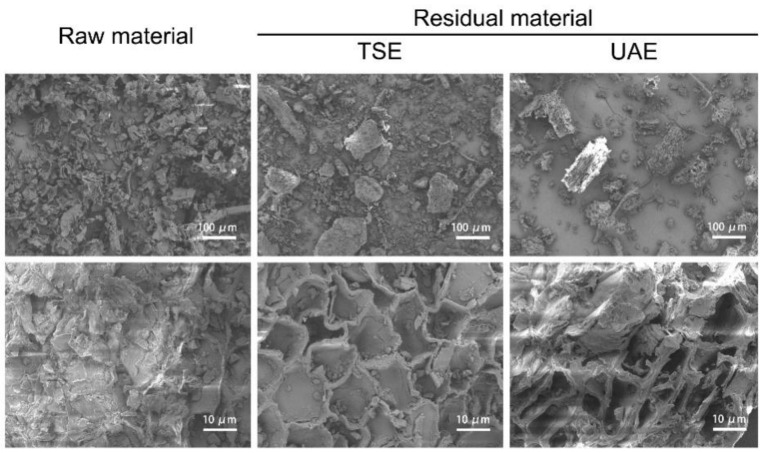
SEM images of *J. mandshurica* powder and its residues treated by UAE and TSE at magnification ×100 and ×1000.

Scanning electron microscopy images of *J. mandshurica* samples and residues treated by different extraction techniques with different magnifications are shown in Figure 6. The surface of the untreated powder was intact, which was obviously different to the surface morphology of the powders treated by the two methods described above. Many holes appeared in the samples treated by UAE or TSE, and the holes were deeper in the powders treated by UAE. It was speculated that this might be due to the characteristics of ultrasound, such as the cavitation phenomena, sponge effect, and mechanical mixing effect. Indeed, ultrasound treatment can generate a certain number of pores and greatly change the structure of plant materials, which is more conducive to the diffusion of extraction solvent into cells and the leakage of intracellular solutes, so as to facilitate the extraction of the effective components [26,27].

#### 2.5.2. FTIR Spectroscopy Analysis of *J. mandshurica*-Derived Flavonoids Obtained Using Different Extraction Techniques

**Figure 7:**
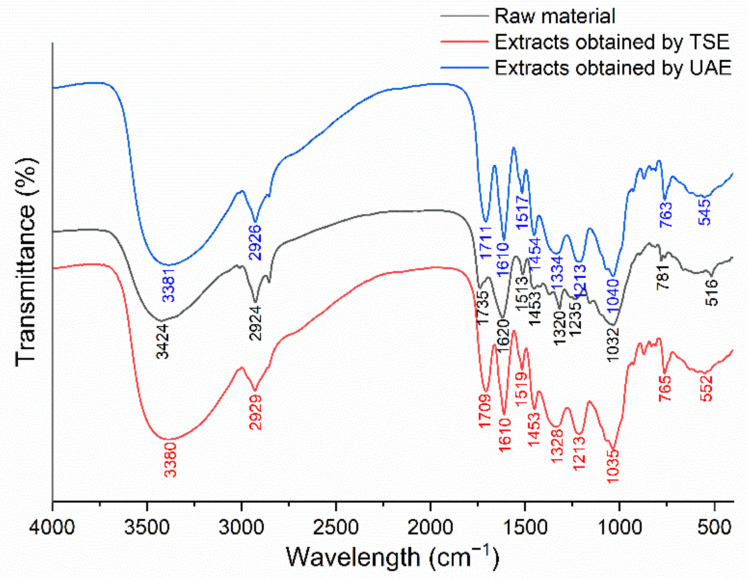
FTIR spectrum analysis of the raw materials and extracts obtained by UAE and TSE.

The FTIR spectra of the *J. mandshurica* powder and extracts obtained using different extraction techniques are presented in Figure 7. The spectra of three samples displayed a number of absorption bands, indicating the complex nature of the tested materials. The spectra of the raw material and extracts obtained by TSE and UAE showed wide characteristic absorption bands at 3424, 3380, and 3381 cm^−1^, respectively, which could have been due to the stretching of the O–H group. FTIR absorption at 2924, 2929, and 2926 cm^−1^ may have been caused by stretching vibrations of the C–H group (2950–2850 cm^−1^). The bands at 1735, 1709, and 1711 cm^−1^ represented the stretching vibrations of the C=O group (1750–1700 cm^−1^). Additionally, other assignments of functional groups responsible for FTIR absorption were as follows: 1213, 1235 cm^−1^ (C–O stretch), 1032, 1035, and 1040 cm^−1^ (C–C stretch). All the absorptions identified above may be related to the presence of flavonoids [28]. Furthermore, the extracts obtained by TSE and UAE showed similar spectra, indicating that the functional groups of the extracted compounds did not change in the process of ultrasonic treatment, which further confirmed the feasibility of ultrasonic extraction technology.

### 2.6. Antioxidant Activity of J. mandshurica-Derived Flavonoids

**Figure 8:**
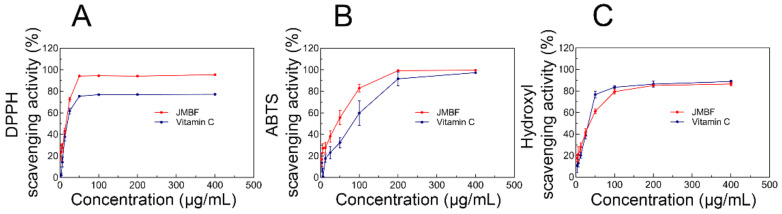
In vitro antioxidant capacity of flavonoids derived from *J. mandshurica*. (**A**) DPPH-scavenging activity of JMBF. (**B**) ABST radical-scavenging activity of JMBF. (**C**) Hydroxyl radical-scavenging activity of JMBF.

Recently, it has been shown that excessive formation of free radicals contributes to the oxidative stress involved in many pathological states and diseases, especially circulatory, respiratory, and nervous systems diseases [29,30]. Antioxidants, also known as “free-radical scavengers”, are the substances that inhibit oxidation [31]. Thus, a large amount of research has focused on the potential use of exogenous antioxidants to reduce oxidative stress [32]. Food and medicinal plants are rich in polyphenols, vitamins, and other components and are considered the main sources of natural antioxidants [33]. For assessing the antioxidant ability of the flavonoids obtained by UAE, a variety of in vitro tests were carried out in this study. As illustrated in Figure 8A, JMBF exhibited a positive effect on the DPPH free-radical scavenging capacity. The DPPH radical-scavenging rate of 5 mg·mL^−1^ JMBF reached 80%, which was equivalent to the positive control drug (Vc). Similarly, JMBF also proved to have strong ABTS^+^ and hydroxyl radical-scavenging ability (Figure 8B,C). The above data confirmed the antioxidant potential of *J. mandshurica*-derived flavonoids, which proved, together with the results of previous studies, that flavonoids exert beneficial effects on oxidation. Kamiyama et al. demonstrated that flavonoids found in young green barley leaves may prevent diseases caused by oxidative damage [34]. It was reported that naringin and its aglycone naringenin, belonging to this series of flavonoids, were found to display strong antioxidant activity [35]. These results further promote the development and utilization of plant flavonoids.

## 3. Materials and Methods

### 3.1. Material

The bark of *J. mandshurica* was collected in July at Panshi, Jilin Province, China. Samples were dried in the oven (DHG-9076A, Jinghong, Shanghai, China) at 50 °C and ground to a fine powder (60 mesh) using a grinder (Taisite, Tianjin, China). The powder was stored in an airtight container for further use.

### 3.2. Optimization of UAE Conditions of Flavonoids from J. mandshurica

#### 3.2.1. Extraction Procedures

JMBF was extracted from *J. mandshurica* using UAE. Briefly, powdered material (0.5 g) was mixed with an appropriate volume of ethanol solvent in a 50 mL tube and put into the ultrasonic bath (KunShan Ultrasonic Instruments Co., Ltd., KQ-250DB, Suzhou, China) for a certain time. The extracted mixture was centrifuged (8000× *g*, 10 min), and the content of flavonoids in the supernatant was detected.

#### 3.2.2. Single-Factor Test

For investigating the effect of various ethanol concentrations (30%, 40%, 50%, 60%, 70%, and 80%), ultrasonic power (125, 150, 175, 200, 225, and 250 W), extraction temperatures (30, 40, 50, 60, 70, and 80 °C), solid–liquid ratios (1:10, 1:15, 1:20, 1:25, 1:30, and 1:35 g·mL^−1^), extraction times (20, 30, 40, 50, 60, and 70 min), and extraction times (1, 2, 3, 4, and 5) on the extraction yield of flavonoids, a single-factor test was employed.

#### 3.2.3. Plackett–Burman Design Experiments

To determine the main factors affecting the extraction yield of JMBF, an eight-factor two-level PBD was introduced. The levels of the selected factors and the design matrix of PBD are listed in Table A2 and Table 1, respectively. A first-order polynomial equation was established to describe the relationship between the selected factors and the response variable [36]. Furthermore, the obtained results were subjected to an analysis of variance, which was carried out using SAS V8 software (Institute Inc., Cary, NC, USA). Meanwhile, a Pareto plot was drawn by Minitab 15 software (Minitab Inc., State College, PA, USA) to further intuitively analyze the importance of the investigation factors on the response variable.

#### 3.2.4. Box–Behnken Design Experiments

For obtaining the most suitable value for the main factors, a BBD design with three factors and 15 runs was generated by Design-Expert 11 software (Stat-Ease Inc., Minneapolis, MN, USA). The ranges of the tested main variables (extraction temperature X_1_, ethanol concentration X_2_, and ultrasonic power X_3_) and experimental runs are given in Table 2. To determine the optimal extraction conditions, response surface methodology (RSM) and ANN–GA were employed to analyze the results. In RSM, a quadratic regression model was introduced using Design-Expert 11 software to evaluate the linear and interactive effects of different factors on the response variable [37]. An analysis of variance (ANOVA) was carried out, and the effect and regression coefficients of individual linear, quadratic, and interaction terms were determined. Response surfaces and contour plots were also drawn to intuitively understand the interactive effects of the various factors.

Furthermore, feedforward ANN backpropagation was adopted for the dynamic modeling of the extraction process using MATLAB software (MATLAB R2009a, MathWorks Inc., Natick, MA, USA), which was composed of multiple interconnected artificial neurons and grouped to form three basic units, namely, input layer, hidden layer, and output layer [38]. Among them, 11 samples were used for training, two samples were used for validation, and two samples were used for testing. In the present work, the ANN was trained using the Levenberg–Marquardt backpropagation algorithm. Considering that the number of hidden nodes has a serious impact on the performance of the ANN model, the degree of approximation (*Da*) was introduced to screen the best value of this number according to the literature method [39]. The calculation formula of *Da* was as follows:

(4) $$D_{a}=\frac{c}{\frac{n_{c}}{n}\times RMSE_{c}+\frac{n_{t}}{n}\times RMSE_{t}+\left|RMSE_{c}-RMSE_{t}\right|}$$

where *n* represents the total number of the calibration set and test set, *n_c_* and *n_t_* are the number of the calibration set and test set, and *RMSE_c_* and *RMSE_t_* are the root-mean-square error (*RMSE*) of the calibration set and test set, respectively; the constant *c* was set to 3.

Once an optimal ANN model was established, GA was implemented referring to the literature method to predict the maximum responding value and the best value for each factor [40]. The parameters of GA optimization (population type, double vector; population size, 20; initial population, given randomly; selection function, stochastic uniform; elite count, 2; crossover fraction, 0.8; crossover function, scattered; migration fraction, 0.2; migration interval, 20) were established. Optimum extraction conditions were selected after evaluation of GA for 100 epochs in the given range of input parameters.

#### 3.2.5. Verification Test

JMBFs were extracted five times from *J. mandshurica* using the obtained optimal extraction conditions, and the average amount of flavonoids was calculated.

#### 3.2.6. Analysis of Flavonoid Amount in Extracts

The content of flavonoids was determined by the NaNO_2_–Al(NO_3_)_3_–NaOH colorimetric method, and rutin was introduced as the standard substance [41]. An amount of 200 μL extract was well mixed with NaNO_2_ (5%, *m*/*v*, 500 μL) and Al (NO_3_)_3_ (10%, *m*/*v*, 500 μL). The mixture was kept for 6 min, followed by the addition of a certain amount of NaOH (1 M, 3 mL). The volume of the above reaction system was fixed to 10 mL with an ethanol solution (60%, *v*/*v*), and it was left at room temperature for 15 min. The absorbance at 510 nm was determined by spectrophotometer (T600, Persee, Beijing, China), and the content of the total flavonoids was calculated.

### 3.3. Traditional Solvent Extraction (TSE)

An ethanol reflux extraction was also introduced for the preparation of JMBF. The extraction conditions were as follows: concentration of extraction solvent, 60%; temperature, 60 °C; solid–liquid ratio, 1:20 g·mL^−1^; extraction time, 40 min. The supernatant was then harvested, and the amount of flavonoid in the extract was determined.

### 3.4. Study on Kinetics of Batch Extraction

To explore the kinetics of two different extraction techniques, JMBF was extracted in batches using the obtained optimal UAE and TSE process described above. After extraction for different times, the extracts were harvested, and the content of the flavonoids was detected. The dissociation rate of JMBF was expressed by the following formula [42]:

(5) $$r=\frac{dC}{dt}=k{\left(C_{S}-C_{t}\right)}^{n},$$

where *t* represents the extraction time (min). *C_t_* and *C_s_* denote the concentration of flavonoids at time *t* and the saturated concentration of flavonoids, respectively. The rate of extraction, the rate constant for extraction, and order of reaction are represented by *r*, *k*, and *n*, respectively.

### 3.5. Scanning Electron Microscopy (SEM)

A ZEISS Gemini Sigma 300 microscope (ZEISS, Oberkochen, Germany) was used for the SEM analysis as per a previous study. Images were acquired in low vacuum at an operating voltage of 5 kV.

### 3.6. Fourier-Transform Infrared Spectroscopy (FTIR)

In this analysis, the dehydrated flavonoids obtained by UAE and TSE were mixed with a certain amount of potassium bromide and scanned using an FTIR spectrometer (Thermo Nicolet IS 50, Thermo Fisher, Waltham, MA, USA). The infrared spectrum of the samples within the region of 4000–400 cm^−1^ was obtained [43].

### 3.7. Antioxidant Activity of the J. mandshurica-Derived Flavonoids

The antioxidant potential of JMBF extracted by UAE was evaluated via multiple in vitro tests as described previously, including the scavenging assays of DPPH, ABTS, and hydroxyl radical [44,45,46]. Vc was introduced as a positive control substance.

Aliquots of extracts (10–100 μL) or standard solution of vitamin C (Vc) were added into ethanol (0.9 mL). Then, 0.6 mL of the above solution was taken and fully mixed with 2.4 mL of DPPH reagent (0.4 mmol/L). The mixture was placed in the dark to react for 40 min, and the absorbance at 520 nm was determined using a Microplate reader (Spark, Tecan, Männedorf, Switzerland). The DPPH radical-scavenging activity of the sample was calculated.

To determine the ABTS radical-scavenging ability of JMBF, 7 mmol/L ABTS solution was mixed with 2.5 mM potassium persulfate (final concentration), and was allowed to react at room temperature and away from light for 16 h to generate ABTS radical cations (ABTS^+^). The ABTS^+^ solution was diluted with methanol until the absorbance reached 0.70 at 730 nm. Then, aliquots of samples (10 to 100 µL) or appropriate amounts of Vc standards and ABTS working solution (100 µL) were vigorously mixed, and the mixture was left for 20 min in the dark at room temperature. The absorbance at 730 nm was measured.

In the scavenging assays of hydroxyl radical, 1 mL of FeSO_4_ (10 mmol/L), 1 mL of H_2_O_2_ (8 mmol/L), and 0.8 mL of sample were well mixed, and then 1 mL of salicylic acid (10 mmol/L) was added. The mixture was incubated in a water bath at 37 °C for 1 h, and its absorbance at 510 nm was determined.

### 3.8. Statistical Analysis

Data obtained from all experiments repeated at least three times were expressed as the mean ± standard deviation (SD).

## 4. Conclusions

In the present study, the ultrasonic-assisted extraction technique was successfully used in the extraction of flavonoids from *J*. *mandshurica*. The suitable conditions optimized by artificial intelligence-based tools were as follows: ethanol concentration, 62%; ultrasonic power, 228 W; solid–liquid ratio, 1:20 g·mL^−1^; extraction temperature, 60 °C; extraction time, 40 min; total number of extraction times, SEM images showed that the structure of the plant materials changed markedly under the action of ultrasound, and the synergistic action of the ultrasound and solvents was conducive to the dissolution of the active substances. Meanwhile, the results of FTIR spectroscopy showed that ultrasound did not change the functional groups in the extracts. Furthermore, the antioxidant potential of flavonoids extracted by UAE was confirmed. Overall, these data demonstrate that ultrasonic technology provides a reference for effective extraction technology for natural antioxidant products.

## Figures and Tables

**Table 1:** Design matrix and results of PBD experiment.

Run	X_1_	X_2_	X_3_	X_4_	X_5_	X_6_	X_7_	X_8_	Y (mg·g^−1^)
1	−1	−1	−1	1	1	1	−1	1	3.65 ± 0.33
2	−1	−1	−1	−1	−1	−1	−1	−1	4.29 ± 0.14
3	1	1	−1	1	1	−1	1	−1	4.26 ± 0.24
4	−1	1	−1	−1	−1	1	1	1	5.41 ± 0.14
5	1	−1	−1	−1	1	1	1	−1	5.87 ± 0.17
6	−1	1	1	−1	1	−1	−1	−1	6.16 ± 0.11
7	1	1	1	−1	1	1	−1	1	5.95 ± 0.14
8	1	1	−1	1	−1	−1	−1	1	5.74 ± 0.14
9	1	−1	1	−1	−1	−1	1	1	4.91 ± 0.35
10	−1	1	1	1	−1	1	1	−1	4.44 ± 0.09
11	−1	−1	1	1	1	−1	1	1	5.39 ± 0.06
12	1	−1	1	1	−1	1	−1	−1	4.76 ± 0.24

**Table 2:** Design matrix and results of BBD experiment.

Run	Extraction Temperature (X_1_, °C)	Ethanol Concentration (X_2_, %)	Ultrasonic Power (X_3_, W)	Extraction Rate (Y, mg·g^−1^)
1	50 (−1)	40 (−1)	225 (0)	5.32 ± 0.11
2	50 (−1)	80 (1)	225 (0)	5.63 ± 0.13
3	70 (1)	40 (−1)	225 (0)	5.49 ± 0.17
4	70 (1)	80 (1)	225 (0)	6.04 ± 0.14
5	60 (0)	40 (−1)	200 (−1)	4.96 ± 0.22
6	60 (0)	40 (−1)	250 (1)	4.99 ± 0.14
7	60 (0)	80 (1)	200 (−1)	5.49 ± 0.15
8	60 (0)	80 (1)	250 (1)	5.56 ± 0.06
9	50 (−1)	60 (0)	200 (−1)	5.40 ± 0.09
10	70 (1)	60 (0)	200 (−1)	5.29 ± 0.17
11	50 (−1)	60 (0)	250 (1)	5.29 ± 0.18
12	70 (1)	60 (0)	250 (1)	5.38 ± 0.08
13	60 (0)	60 (0)	225 (0)	6.15 ± 0.05
14	60 (0)	60 (0)	225 (0)	6.24 ± 0.03
15	60 (0)	60 (0)	225 (0)	6.18 ± 0.12

**Table 3:** Analysis of variance for MQR model.

Source	DF	SS	MS	F	Pr > F
X_1_	1	0.0390	0.0390	3.2752	0.1301
X_2_	1	0.4831	0.4831	40.5320	0.0014
X_3_	1	0.0008	0.0008	0.0638	0.8107
X_1_ × X_1_	1	0.2111	0.2111	17.7148	0.0084
X_1_ × X_2_	1	0.0143	0.0143	1.1986	0.3235
X_1_ × X_3_	1	0.0100	0.0100	0.8414	0.4011
X_2_ × X_2_	1	0.3973	0.3973	33.3355	0.0022
X_2_ × X_3_	1	0.0004	0.0004	0.0365	0.8561
X_3_ × X_3_	1	1.3804	1.3804	115.8228	0.0001
Model	9	2.3317	0.2591	21.7385	0.0017
(Linear)	3	0.5229	0.1743	14.6236	0.0066
(Quadratic)	3	1.7841	0.5947	49.8997	0.0004
(Cross-product)	3	0.0247	0.0082	0.6922	0.5951
Error	5	0.0596	0.0119		
(Lack of fit)	3	0.0552	0.0184	8.3424	0.1089
(Pure error)	2	0.0044	0.0022		
Total	14	2.3913			

## Data Availability

Data presented in the present study are available on request from the corresponding author.

## Sample Availability

Samples of the compounds are available from the authors.

## Appendix A

**Table A1:** Analysis of variance for PBD experiment.

Source	DF	SS	MS	F	Pr > F
X_1_	1	1.1312	1.1312	17.9156	0.0241
X_2_	1	4.3681	4.3681	69.1791	0.0036
X_3_	1	0.0081	0.0081	0.1283	0.7439
X_4_	1	0.8740	0.8740	13.8424	0.0338
X_5_	1	0.2679	0.2679	4.2429	0.1315
X_6_	1	0.1347	0.1347	2.1327	0.2403
X_7_	1	0.0296	0.0296	0.4694	0.5424
X_8_	1	0.0536	0.0536	0.8485	0.4249
Model	8	6.8673	0.8584	13.5949	0.0275
Error	3	0.1894	0.0631		
Total	11	7.0567			

**Table A2:** Levels of factors selected for use in the PBD experiment.

Level	Factors				
	Ethanol Concentration (X_1_, %)	Extraction Temperature (X_2_, °C)	Extraction Time (X_3_, min)	Ultrasonic Power (X_4_, W)	Solid–Liquid Ratio (X_5_, g·mL^−1^)
−1	40	50	40	200	1:10
1	70	75	60	250	1:30
